# Biochemical Characterization of LsGajA: A Key Nuclease for Gabija Defense in Lactic Acid Bacteria

**DOI:** 10.3390/microorganisms14061353

**Published:** 2026-06-16

**Authors:** Kexin Li, Yujing Tian, Juyue Luo, Shiyu Ma, Jinhai Huang, Lei Zhang, Deping Hua

**Affiliations:** School of Life Sciences, Tianjin University, Tianjin 300072, China; 13920967339@163.com (K.L.); tianyujingw@tju.edu.cn (Y.T.); ljyjy525@163.com (J.L.); mashiyu@tju.edu.cn (S.M.); jinhaih@tju.edu.cn (J.H.)

**Keywords:** Gabija system, LsGajA, nuclease, defense mechanisms, lactic acid bacteria

## Abstract

Lactic acid bacteria (LAB), as important probiotics, face challenges in applications from bacteriophage infection and the instability of foreign genetic elements. Although Gabija systems and their GajA nuclease components have been characterized in model bacteria, their distribution, biochemical properties, and defensive functions in LAB remain largely unexplored. Here, we provide the first systematic characterization of a naturally occurring Gabija system from *Ligilactobacillus salivarius* Ren and clarify its distribution among LAB. Approximately 9.3% of LAB strains encode the Gabija system, which exists as a *gajA*-*gajB* gene cluster. We found that the Gabija system originated independently in different bacterial lineages. The GajA of *L. salivarius* Ren (LsGajA) was purified and exhibited non-specific nuclease activity that could efficiently cleave various nucleic acid substrates, including plasmids and linear double-stranded DNA (dsDNA). This activity displayed a temperature-dependent profile, with high activity observed from 45 to 60 °C and at pH 8.0. Mg^2+^ markedly enhanced its degradative nuclease activity, whereas high concentrations of dNTPs inhibited DNA cleavage. LsGajA exhibited substrate-dependent differences in cleavage efficiency, indicating that substrate origin and associated physicochemical features may influence its activity. Additionally, we demonstrate that LsGajA exhibits exceptional stability as a nuclease, retaining activity under a wide range of conditions. The LsGabija system significantly enhanced the ability to reject foreign plasmids and provided strong resistance to the bacteriophage T5 in *Escherichia coli*. This study provides the first systematic biochemical and functional characterization of the Gabija system in LAB, advancing our understanding of this prokaryotic defense system and highlighting its potential for industrial applications.

## 1. Introduction

Lactic acid bacteria (LAB) are Gram-positive microorganisms that primarily ferment carbohydrates into lactic acid and possess a broad spectrum of probiotic functions, including modulation of gut microbiota composition, inhibition of pathogenic microorganisms [1,2], stimulation of immune responses, antitumor activity [3], and antioxidant effects [4]. *Ligilactobacillus salivarius* (formerly named *Lactobacillus salivarius*) was first isolated from the human oral cavity in 1953 [5] and has been identified as a predominant species in the human gastrointestinal tract and breast milk. It is a prominent species widely utilized in the food industry and plays a crucial role in the production of fermented foods, such as yogurt, cheese, sauerkraut, and fermented beverages, by enhancing shelf-life, improving sensory profiles, and contributing to the health-promoting attributes of the products [6]. In recent years, *L. salivarius* has been shown to contribute to maintaining intestinal homeostasis by regulating gut microbiota, enhancing host immunity, and preventing inflammatory disorders [7]. Owing to these probiotic properties, *L. salivarius* has been extensively studied for its applications in food fermentation, as well as in promoting gut health and overall well-being [8,9].

Prokaryotes have evolved various defense systems to resist the invasion of bacteriophages and foreign genetic elements, such as plasmids and transposons [10,11,12]. These systems are crucial for maintaining genomic integrity, restricting horizontal gene transfer, and preventing the invasion of exogenous nucleic acids. Classical defense mechanisms are generally classified into four categories: the restriction-modification (RM) [13], abortive infection (Abi) [14], toxin–antitoxin (TA) [15], and CRISPR-Cas system [16]. Recent advances revealed numerous previously uncharacterized bacterial defense systems [17,18]. These defense strategies enable bacteria to counteract foreign threats and safeguard the survival and persistence of their populations [19].

In the Gabija defense system, it was recently identified that in more than 15% of sequenced bacterial and archaeal genomes [17,20], there are two core genes, *gajA* and *gajB*, whose molecular functions have been partially delineated in *Bacillus cereus* VD045. GajA is a Mg^2+^-dependent, sequence-specific DNA-nicking endonuclease, whose activity is inhibited by high concentrations of nucleoside triphosphates (NTPs and dNTPs) [21,22]. GajB, predicted to be a UvrD/PcrA/Rep-like helicase [17,23], associates with GajA to form a stable GajA-GajB complex [24], which enables efficient defense against phage infection in *B. cereus* VD045 [13]. Moreover, heterologous expression of this system in *Bacillus subtilis* confers resistance to a broad range of phages, including the phages phi29, rho14, phi105, and SPβ [17]. The potent DNA-degrading activity and autoregulatory features of the Gabija system are thought to play critical roles in phage resistance. However, whether Gabija homologs in LAB exhibit conserved or lineage-specific biochemical and defensive properties remains largely unknown, although phage resistance and genetic stability are critical for the performance of probiotic and industrial LAB strains.

In this study, we first analyzed the distribution, genetic organization, and evolutionary features of Gabija systems in LAB. The GajA from *L. salivarius* Ren (hereafter defined as LsGajA) was purified and displayed broad non-sequence-specific nuclease activity against diverse plasmid and linear dsDNA substrates, substrate-dependent differences in DNA cleavage efficiency, dNTP-mediated inhibition, and remarkable thermal and storage stability in vitro. We systematically assessed the influence of temperature, metal ions, pH, and dNTP concentration on the nuclease activity of LsGajA, along with its stability. Furthermore, heterologous expression of LsGajA in *Escherichia coli* was shown to inhibit both plasmid transformation and bacteriophage infection. These findings provide new insights into Gabija-mediated defense in LAB and suggest potential applications in probiotic and industrial biotechnology.

## 2. Materials and Methods

### 2.1. Strains and Plasmids

*L. salivarius* Ren that was isolated from centenarians from Bama in China with a 6× loading buffer [25] was obtained from the Key Laboratory of Functional Dairy at China Agricultural University and maintained at −80 °C. The *E. coli* strains Top10, JM109, DH5α, and BL21 (DE3), maintained in our laboratory, were used for cloning and protein expression.

pET-28a was used for His-tagged LsGajA expression, p15A-Cm-repDE and p15A-Cm-repDE-GajA/B were used as the vector control and Gabija-expression plasmid, respectively, and pOT-RFP was used as the exogenous plasmid challenge. Other plasmids, including F-1315, pJW, pUC19, pBTs, and pGM-T, were used as DNA substrates or cloning vectors for nuclease activity and cleavage-site analyses. Information on the plasmids is listed in Table S1.

### 2.2. Genomic and Phylogenetic Analysis

Antiviral defense systems previously identified were analyzed from 1344 LAB genomes using DefenseFinder [26]. Genomic sequences of strains harboring the Gabija system were retrieved from NCBI. *gajA* and *gajB* were annotated using the NCBI Genome Browser and SnapGene to determine genomic coordinates, orientation, and intergenic or overlapping features. Conserved domains of LsGajA and LsGajB were predicted using the Pfam database based on their amino acid sequences.

The gene clusters of *Gabija* (including *gajA* and *gajB*) were aligned using MEGA 11. Phylogenetic analysis was performed using the neighbor-joining method. Bootstrap support was calculated based on 1000 replicates.

### 2.3. Cloning of the Gabija System

Genomic DNA of *L. salivarius* Ren was extracted using an alkaline lysis method and served as the template for PCR amplification of the *LsGajA-LsGajB* operon. The p15A-Cm-repDE vector was linearized by inverse PCR, and the purified PCR products (Vazyme, Nanjing, China) were assembled using Gibson cloning (Yeasen, Shanghai, China). The assembly mixture was transformed into *E. coli* Top10 competent cells that were cultured overnight at 37 °C and screened by colony PCR. Positive clones were verified by Sanger sequencing and designated p15A-Cm-repDE-GajA/B. The recombinant strain was entitled *E. coli* DE-GajAB and stored in 20% glycerol at −80 °C.

For constructing the expression vector of LsGajA, the coding sequence was amplified from p15A-Cm-repDE-GajA/B and inserted into *SacI*-linearized pET-28a, which served as the empty expression vector, using Gibson assembly (GenStar, Beijing, China). Positive clones were verified by PCR and DNA sequencing (GENEWIZ, Suzhou, China), and the resulting recombinant plasmid was designated pET-28a-GajA. The primer sequences used in this study are listed in Table S2. The nucleotide sequences of *LsGajA* and *LsGajB* from *L. salivarius* Ren are provided in Data S1.

### 2.4. Expression and Purification of Recombinant LsGajA

The plasmid pET-28a-GajA was transformed into *E. coli* BL21(DE3). A single colony was inoculated into LB broth (Kan^+^) and cultured at 37 °C with shaking until OD_600_ ≈ 0.6. Protein expression was induced with 0.5 mM of IPTG at 16 °C for 16 h. Then bacteria were harvested by centrifugation and disrupted using an ultransonic cell disruptor (Scientz, Ningbo, China) in a buffer containing PMSF. The lysate was centrifuged at 18,000 rpm for 50 min at 4 °C, and the supernatant was collected.

The soluble His-tagged LsGajA was purified by Ni-NTA affinity chromatography (Solarbio, Beijing, China) under native conditions without denaturation. The column was washed with a wash buffer (20 mM of Tris-HCl, pH 8.0, 300 mM of NaCl, and 20 mM of imidazole), and protein was eluted with an elution buffer (20 mM of Tris-HCl, pH 8.0, 300 mM of NaCl, and 250 mM of imidazole). Elution fractions were analyzed by SDS-PAGE and Coomassie Brilliant Blue staining. Purified LsGajA was aliquoted in the elution buffer and stored at −80 °C.

### 2.5. DNA Cleavage Assay and Enzymatic Characterization

Enzymatic activity analyses were conducted in a 20 µL reaction containing 200 ng of DNA as the substrate (plasmid or linear dsDNA), 20 mM of Tris-HCl (pH 8.0), 1 mM of MgCl_2_, 1 mM of DTT, and 0.5 µM of purified LsGajA at 37 °C [21]. Reactions were terminated with 6× loading buffer and analyzed by agarose gel electrophoresis using an electrophoresis system (Bio-Rad, Hercules, CA, USA). Cleavage kinetics were monitored from 10 to 60 min. To examine whether the host origin of plasmid DNA influences LsGajA-mediated cleavage, F-1315 plasmid DNA was isolated from *E. coli* DH5α, JM109, and Top10 and used as a substrate under identical reaction conditions with Mg^2+^. The optimal temperature was determined to be between 16 and 65 °C. Effects of ions (Mg^2+^, Mn^2+^, Ca^2+^, Na^+^, and K^+^) at 1 mM were tested, and Mg^2+^ concentration dependence (0.2–100 mM) was evaluated. Reactions were also conducted at pH 8.0, 9.0, and 10.0. Nucleotide inhibition assays were conducted using 1 mM of dATP, dTTP, dCTP, or dGTP, and dATP dose–response curves (0.05–1.0 mM) were generated. Control reactions without LsGajA were included in all assays. Gel band intensities were quantified using ImageJ (v 1.53) [27], and cleavage efficiency was calculated as product intensity/(product + substrate intensity). Data represent means ± SEM from three independent experiments. Statistical analyses and figures were generated using GraphPad Prism (v 10.0).

### 2.6. Determination of DNA Cleavage Sites

The pJW plasmid was divided into three overlapping fragments—dspJW1 (389–3764 bp), dspJW2 (3521–8319 bp), and dspJW3 (7999–765 bp). The F-1315 plasmid (4904 bp) was amplified as two overlapping fragments, dsF-1315F (2305–208 bp) and dsF-1315R (1–2640 bp). Each fragment (200 ng) was incubated with LsGajA under standard conditions (37 °C; 1 mM of MgCl_2_). Reactions were terminated with a 6× loading buffer on ice and analyzed by agarose gel electrophoresis.

For mapping cleavage sites, digested F-1315R fragments were poly(A)-tailed and cloned into pGM-T. Recombinant constructs were transformed into *E. coli* Top10 and grown overnight. Colonies were screened by PCR using M13 universal primers. Clones displaying polymorphic insert sizes were sequenced [27], and cleavage sites were identified by alignment with the reference sequence.

### 2.7. Stability Assessment of LsGajA

Thermal stability was assessed by preincubating the enzyme LsGajA at 65 °C, 80 °C, or 98 °C for 20 min, followed by standard activity assays. For storage stability, LsGajA was kept at 25 °C or 37 °C for 24 h, 48 h, or 7 days before activity assays. Freeze–thaw stability was evaluated after 5, 10, or 20 cycles (−80 °C to ice), and remaining activity was determined under standard conditions.

### 2.8. Evaluation of Gabija-Mediated Resistance to Exogenous Plasmids

To evaluate plasmid defense activity, *E. coli* strains carrying either p15A-Cm-repDE or p15A-Cm-repDE-GajA/B were streaked on LB agar containing chloramphenicol and incubated overnight at 37 °C. Single colonies were made competent following a standard protocol [28]. The exogenous plasmid pOT-RFP, which carries an ampicillin resistance marker, was transformed into both strains. After recovery, transformants were plated on LB agar containing both ampicillin and chloramphenicol to select cells that had acquired pOT-RFP while retaining the resident p15A-Cm-repDE or p15A-Cm-repDE-GajA/B plasmid. Plates were incubated overnight, and transformation efficiency was calculated by counting colony-forming units.

### 2.9. Evaluation of Gabija-Mediated Antiphage Activity

Phage plaque assays were performed to evaluate Gabija-mediated antiphage activity. T5 and T7 phage stocks were mixed with *E. coli* BL21(DE3) cultures grown to OD_600_ ≈ 0.6 at a ratio of 1:1 in an LB medium and incubated at 37 °C for approximately 5 h for phage amplification. Cultures were centrifuged at 12,000 rpm for 2 min, and the supernatant was collected as phage lysate. Phage titers (PFU/mL) were determined using the micro-plaque assay as described previously [29,30]. *E. coli* BL21(DE3) carrying the Gabija-expression plasmid was cultured to OD_600_ ≈ 0.6. A 200 µL culture was mixed with 4 mL of LB top agar (0.75%) at 45 °C and poured onto LB agar plates. Serial dilutions of phage lysates (10^0^–10^−7^) were spotted (1 µL) onto the bacterial lawn. After drying, plates were incubated overnight at 37 °C, and plaques were imaged the following day.

## 3. Results

### 3.1. The Features and Distribution of the Gabija System in LAB

**Figure 1:**
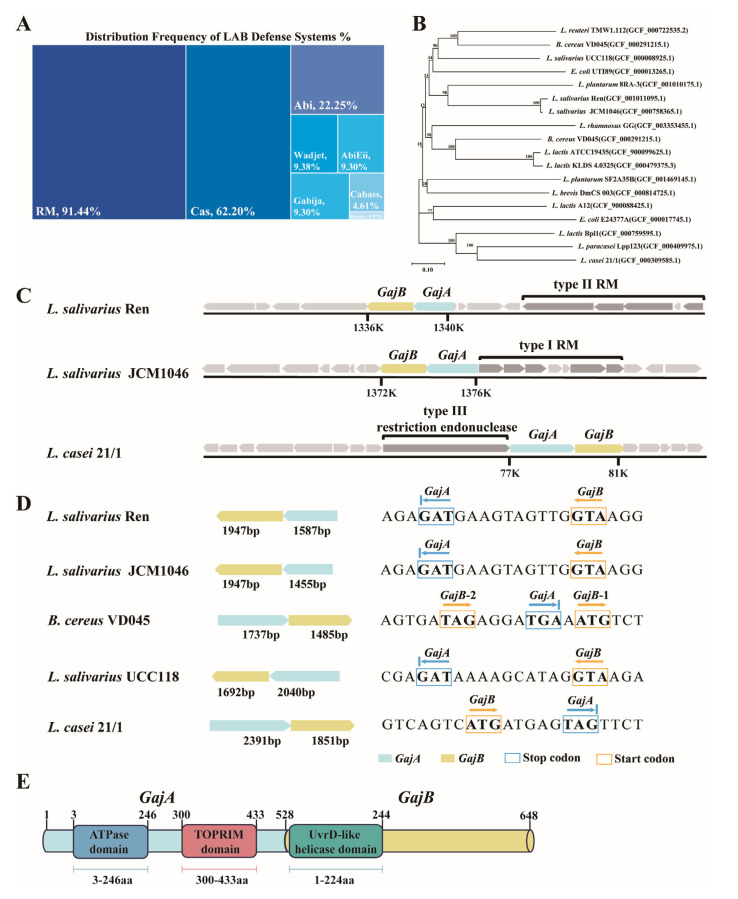
Distribution, phylogeny, genetic organization, and domain of Gabija systems in LAB. (**A**) Distribution frequency of defense systems across 1344 LAB genomes. (**B**) Phylogenetic analysis of Gabija systems from 18 representative bacterial strains. Bacterial genus abbreviations in the table follow current taxonomic nomenclature. The correspondence between abbreviations and full names is as follows: *L. salivarius* corresponds to *Ligilactobacillus salivarius*; *L. casei* corresponds to *Lacticaseibacillus casei*; *L. paracasei* corresponds to *Lacticaseibacillus paracasei*; *L. reuteri* corresponds to *Limosilactobacillus reuteri*; *L. brevis* corresponds to *Levilactobacillus brevis*; *L. plantarum* corresponds to *Lactiplantibacillus plantarum*; *L. rhamnosus* corresponds to *Lacticaseibacillus rhamnosus*; *L. lactis* corresponds to *Lactococcus lactis*; *B. cereus* corresponds to *Bacillus cereus*; and *E. coli* corresponds to *Escherichia coli*. (**C**) Genomic context of the Gabija system, frequently located adjacent to Type I, II, or III restriction-modification systems in LAB. (**D**) Analysis of the *GajA* and *GajB* junction, with the core genes *GajA* and *GajB* forming a compact cluster. Right, enlarged nucleotide sequences at *GajA–GajB* junctions. Blue and yellow boxes indicate stop codons and start codons, respectively. Arrows above the sequences indicate the transcriptional orientations of *GajA* and *GajB*. In the case of *B. cereus* VD045, GajB contains two start codons, labeled as *GajB-1* and *GajB-2*. (**E**) Domain organization of LsGajA and LsGajB. LsGajA contains a conserved ATPase domain (amino acids 3–246) and a TOPRIM domain (amino acids 300–433), and LsGajB contains a conserved UvrD-like helicase domain (amino acids 1–244).

The Gabija system was detected in 9.30% of the 1344 sequenced LAB genomes using DefenseFinder [26] (Figure 1A). Although its prevalence is substantially lower than that of the RM (91.44%) and CRISPR-Cas (62.20%) systems, it is comparable to that of other defense modules, such as Wadjet (9.38%) [31] and AbiEii (9.30%) [32]. LAB strains harboring Gabija were isolated from diverse ecological niches, including the animal gastrointestinal tract, soil, and fermented dairy products (Table 1), indicating that this system is present across multiple environments.

To analyze the distribution of the Gabija system in bacteria, we constructed a phylogenetic tree based on the 18 homologs from 14 LAB, 2 *B. cereus*, and 2 *E. coli* strains and found that they grouped into different clades without clear separation among their species origin (Figure 1B). For instance, the Gabija system from *L. salivarius* Ren was most closely related to that of *L. salivarius* JCM1046, but it also was clustered with the systems from *B. cereus* VD045 and *E. coli* UTI89. This finding indicates that the Gabija system may be disseminated predominantly through horizontal gene transfer (HGT) rather than vertical inheritance [17,33], explaining its broad phylogenetic distribution. The genomic context carrying these Gabija systems was analyzed. We found that they were frequently located adjacent to the genes encoding Type I, II, or III RM systems in the genome of LAB (Figure 1C). The core genes *gajA* and *gajB* formed a compact cluster separated by only a few nucleotides or were overlapping, indicating potential co-transcription as a single operon (Figure 1D and Figure S1). Further domain analysis revealed that LsGajA contains a conserved ATPase domain (amino acids 3–246) and a TOPRIM domain (amino acids 300–433), while LsGajB contains a conserved UvrD-like helicase domain spanning amino acids 1–244 (Figure 1E). Although no canonical conserved domain was confidently assigned to the C-terminal region of LsGajB, structural homology with the reported GajA-GajB complex models suggests that this region may represent a flexible helicase-associated region [34]. To further support this analysis, AlphaFold-predicted structural models of LsGajA and LsGajB are provided (Figure S2). These conserved domain features suggest that the functional architecture of the Gabija system may be conserved across bacteria.

### 3.2. LsGajA Is a Non-Specific DNA Nuclease

**Figure 2:**
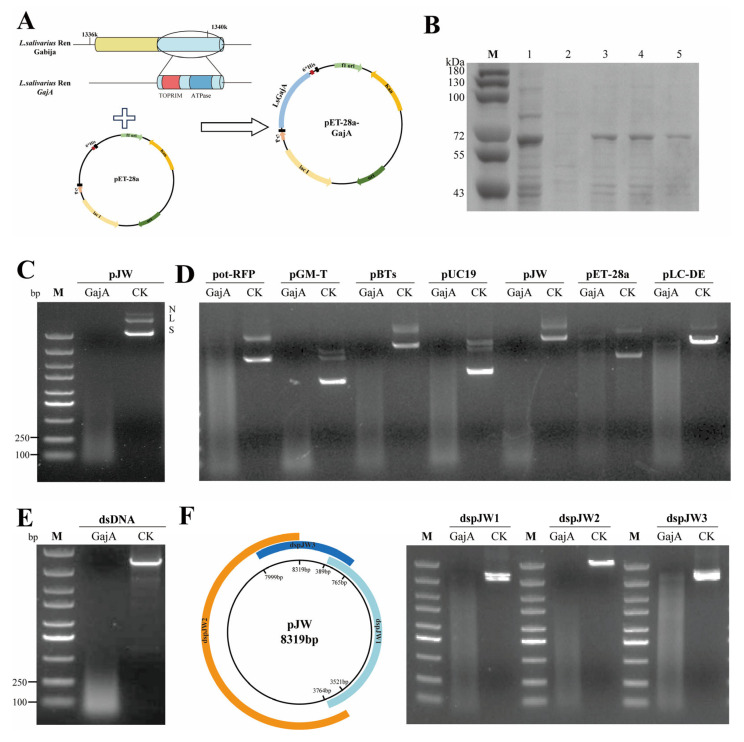
LsGajA is a non-specific nuclease that degrades diverse DNA substrates. (**A**) Schematic of the prokaryotic expression construct containing the *LsGajA* coding sequence with a C-terminal 6 × His tag. (**B**) SDS-PAGE analysis of purified LsGajA. Lane 1: flow-through after binding to the affinity resin; Lane 2: wash fraction containing non-specifically bound proteins; Lane 3: first elution fraction containing the target protein; Lane 4: second elution fraction; Lane 5: third elution fraction. (**C**,**D**) LsGajA degrades distinct plasmids into a DNA smear. “S”, “L”, and “N” indicate the positions of supercoiled, linearized, and nicked circular DNA, respectively. The lanes labeled “CK” represent substrate controls without added LsGajA. All cleavage assays were performed with 1 mM of MgCl_2_ at 37 °C. (**E**) LsGajA degrades linear double-stranded DNA (dsDNA). (**F**) Cleavage of overlapping PCR fragments spanning the pJW plasmid, showing sequence-independent nuclease activity. dspJW1: 389–3764 bp; dspJW2: 3521–8319 bp; and dspJW3: 7999–765 bp.

To investigate the function of GajA in LAB, we amplified *GajA* (*LsGajA*: 1,338,707–1,340,293, from the start codon to stop codon) from *L. salivarius* Ren and constructed a pET-28a-*LsGajA* vector with a C-terminal 6 × His tag (Figure 2A). LsGajA was induced by IPTG and purified in *E. coli* BL21(DE3) (Figure 2B and Figure S3) for functional analysis in vitro.

Given that BcGajA from the Gabija system of *B. cereus* VD045 exhibits DNA cleavage activity [21], we examined whether LsGajA exhibited similar enzymatic activity. LsGajA efficiently degraded the pJW plasmid in all conformations (supercoiled, nicked, and linear), generating random fragments of approximately 100–200 bp (Figure 2C). To further evaluate the substrate spectrum of LsGajA, seven various plasmids, such as pUC19, pET28a, pGM-T and pJW, with distinct backbones were tested. LsGajA could degrade all plasmids into a DNA smear (Figure 2D). Additionally, LsGajA effectively degraded linear double-stranded DNA (dsDNA) (Figure 2E). These data confirm that LsGajA acts as a broad-spectrum nuclease capable of hydrolyzing both plasmid and linear dsDNA.

Because many nucleases exhibit sequence-specific cleavage, we next sought to determine whether LsGajA displayed sequence preference. Three overlapping PCR fragments spanning the entire pJW plasmid (dspJW1: 389–3764 bp; dspJW2: 3521–8319 bp; and dspJW3: 7999–765 bp) were used as substrates. LsGajA degraded all dsDNA into smeared DNA bands regardless of their sequences or length, and no specific cleavage pattern was observed (Figure 2F). Collectively, these findings demonstrate that LsGajA functions as a non-specific nuclease capable of efficiently degrading diverse dsDNA substrates independent of sequence context.

### 3.3. Substrate-Dependent Differences in the Nuclease Activity of LsGajA

**Figure 3:**
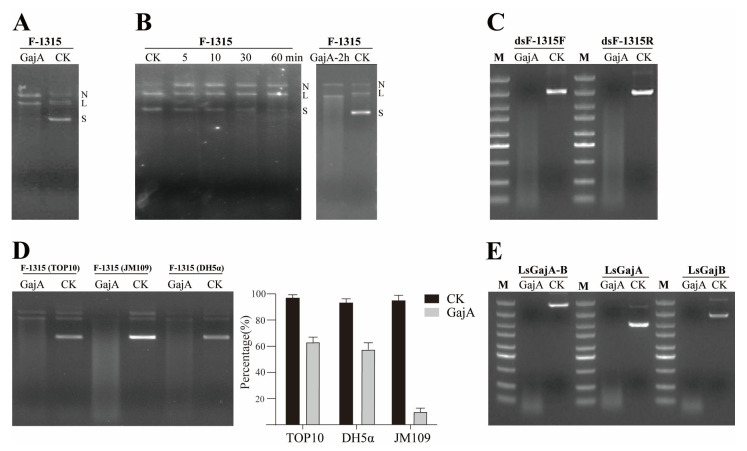
The nuclease activity of LsGajA is modulated by DNA substrates and host-dependent epigenetic modifications. (**A**,**B**) LsGajA cleavage assays using the supercoiled F1315 plasmid. LsGajA rapidly converted the supercoiled form (S) into nicked (N) and linear (L) intermediates (**A**), but further degradation into small fragments was markedly limited, even after extended incubation (5–60 min; 2 h) (**B**). (**C**) Cleavage of two linear PCR fragments amplified from the F1315 plasmid (dsF-1315F and dsF-1315R). (**D**) LsGajA activity on F1315 plasmids isolated from different *E. coli* strains, Top10, JM109, and DH5α. Bar graphs represent the average of three independent experiments, with error bars representing the standard error of the mean. (**E**) Degradation of genomic PCR fragments from *L. salivarius* Ren, including the *LsGajA-B* region, *LsGajA*, and *LsGajB*.

Although LsGajA efficiently degraded various plasmid and linear double-stranded DNA substrates, its cleavage efficiency toward the F1315 supercoiled plasmid was markedly reduced (Figure 3A). Within 5 min, LsGajA cleaved the supercoiled F1315 plasmid into nicked and linear intermediates; however, its ability to further degrade these intermediates was substantially restricted (Figure 3B). Even after 2 h of incubation, a considerable fraction of the nicked and linear forms of F1315 remained, without being fully degraded into small fragments (Figure 3B).

To determine whether intrinsic features of the F1315 sequence contributed to this limited degradation, two linear double-stranded DNA fragments (dsF-1315F: 2305–208 bp and dsF-1315R: 1–2640 bp) were PCR-amplified from the same plasmid and used as substrates. In contrast to the plasmid form, these linear PCR products were readily degraded into small DNA fragments by LsGajA (Figure 3C). Cloning and sequencing of the digested products revealed heterogeneous fragment lengths and randomly distributed cleavage sites, with no detectable conserved sequence motifs (Figure S4; Table S3), indicating that LsGajA acts as a non-specific nuclease with largely random cleavage patterns.

We next examined whether the origin of the DNA substrate affected LsGajA activity. F1315 plasmids isolated from three *E. coli* strains (Top10, DH5α, and JM109) were compared. Notably, the plasmid derived from JM109 was extensively degraded into small fragments, whereas plasmids from Top10 and DH5α predominantly accumulated in nicked and linear forms with limited further degradation (Figure 3D). Given that these plasmids share identical DNA sequences, the observed differences suggest that host-dependent factors influence LsGajA cleavage efficiency.

To further evaluate whether LsGajA discriminates between different DNA sources, linear double-stranded PCR fragments were derived from the *L. salivarius* Ren genome—including the *LsGajA-B* region (genomic coordinates: 1,336,359–1,340,554 bp), *LsGajA* (genomic coordinates: 1,338,677–1,340,554 bp), and *LsGajB* (genomic coordinates: 1,336,360–1,338,987 bp). All fragments were effectively degraded into small DNA products, appearing as typical smears on agarose gels (Figure 3E). This result indicates that LsGajA can efficiently cleave PCR-amplified linear DNA fragments corresponding to its native genomic loci under in vitro conditions. Taken together, these findings indicate that LsGajA-mediated DNA cleavage is influenced by substrate form and DNA host origin. Such variation is unlikely to arise from DNA sequence differences but may instead be associated with host-specific DNA modification patterns or other physicochemical properties acquired by DNA during propagation in distinct *E.coli* hosts.

### 3.4. Determination of the Optimal Reaction Conditions for LsGajA

**Figure 4:**
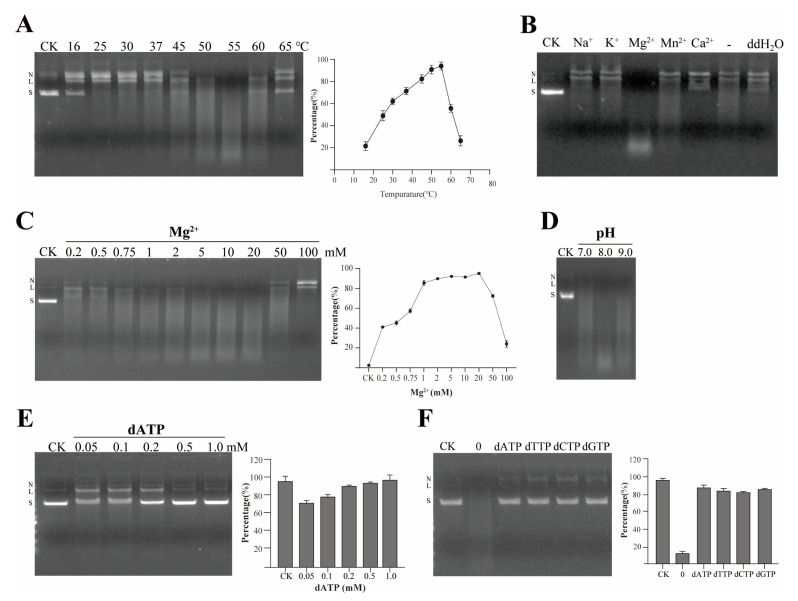
Biochemical characterization and optimal conditions of LsGajA. (**A**) Temperature-dependent cleavage of supercoiled plasmids by LsGajA. (**B**) Effects of different metal ions on LsGajA activity. (**C**) Mg^2+^ concentration dependence. (**D**) pH dependence of LsGajA activity. (**E**,**F**) dNTP inhibition. All four dNTPs, exemplified by dATP (**E**), inhibited LsGajA activity in a dose-dependent manner (**F**). Bar graphs represent the average of three independent experiments, with error bars representing the standard error of the mean (SEM).

To further elucidate the biological function of the Gabija system, we systematically evaluated various factors regulating the nuclease activity of LsGajA, including temperature, metal ion type and concentration, pH, and dNTPs. Within the temperature range of 16–65 °C LsGajA effectively cleaved supercoiled plasmids into nicked and linear forms, accompanied by DNA smearing. The enzymatic activity increased with temperature and then declined, reaching its maximum between 45 and 60 °C, at which LsGajA efficiently degraded plasmids into non-specific DNA fragments (Figure 4A).

The nuclease activity of LsGajA was strongly dependent on divalent metal ions, with an efficacy order of Mg^2+^ > Mn^2+^ ≈ Ca^2+^, while monovalent ions (Na^+^ and K^+^) showed no significant effect (Figure 4B). Notably, LsGajA can still cleave supercoiled plasmids into nicked and linear DNA even in the ddH_2_O reaction system (Figure 4B). A concentration of Mg^2+^ (0.2–100 mM) further revealed that LsGajA exhibited maximal activity at 1–20 mM (Figure 4C). At higher Mg^2+^ concentrations (50–100 mM), LsGajA retained nicking activity but largely lost its ability to further degrade DNA into small fragments, exhibiting activity characteristic of a nicking endonuclease. LsGajA exhibited maximal nuclease activity in the presence of Mg^2+^, whereas Mn^2+^ supported only relatively weak activity. This metal-ion preference differs from the previously reported BcGajA, in which Mn^2+^ strongly stimulates DNA cleavage activity [21,34]. Such differences may be associated with variations in residues located near the predicted metal-binding/catalytic region. Additionally, LsGajA exhibited optimal activity at pH 8.0, at which plasmid degradation was most efficient (Figure 4D).

dATP inhibited LsGajA in a dose-dependent manner (0.05–1.0 mM), with near-complete inhibition at ≥0.5 mM (Figure 4E). dTTP, dCTP, and dGTP exhibited similar inhibitory effects (Figure 4F), indicating that high dNTP concentrations suppress LsGajA function. These findings suggest that intracellular dNTP pools under normal physiological conditions may keep LsGajA in an inhibited state. During the invasion of foreign plasmids or infection by phages, the rapid and massive replication of DNA leads to substantial consumption of local dNTPs. This may temporarily reduce the availability of dNTPs near replication sites, thereby relieving the inhibition of LsGajA and activating its nuclease activity to exert a defensive function.

### 3.5. LsGajA Exhibits Thermal and Storage Stability

**Figure 5:**
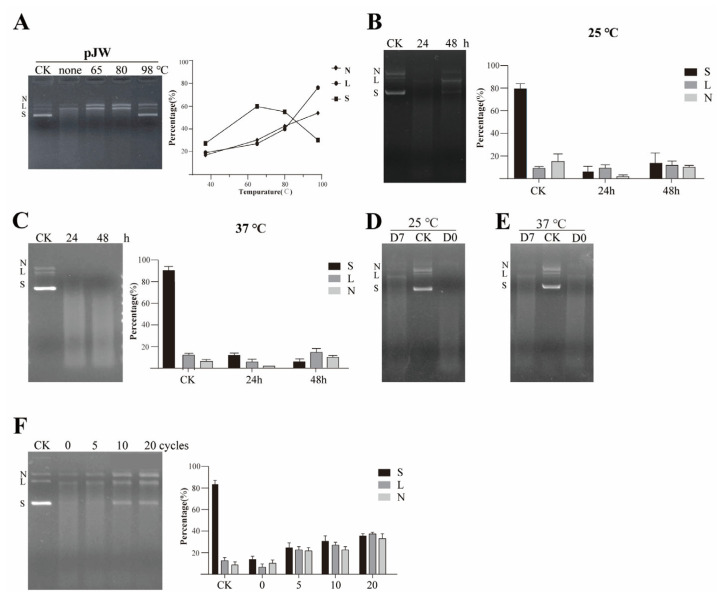
Stability profiling of LsGajA nuclease. (**A**) Thermal stability of LsGajA. Purified protein was preincubated at the indicated temperatures for 20 min, followed by assessment of its ability. (**B**,**C**) Storage stability of LsGajA at 25 °C (**B**) and 37 °C (**C**). Enzyme activities were examined after incubation for 24 h and 48 h. (**D**,**E**) Long-term stability of LsGajA after 7-day incubation at 25 °C (**D**) or 37 °C (**E**). (**F**) Effect of repeated freeze–thaw cycles (0–20 cycles) on LsGajA activity. “S”, “L”, and “N” indicate the positions of supercoiled, linearized, and nicked circular DNA, respectively.

During the investigation of temperature effects on LsGajA, we found that LsGajA retained activity for converting supercoiled plasmids to nicked and linear forms after preincubation at 65 °C and 80 °C for 20 min. Remarkably, even after incubation at 98 °C, LsGajA preserved partial nicking activity (Figure 5A), indicating it has substantial intrinsic thermal stability. *L. salivarius* Ren exhibits relatively high survival rates at 55 °C, which is consistent with the heat-resistant activity of LsGajA and suggests that the Gabija system may contribute to host adaptation under high-temperature stress.

We next assessed the storage stability of LsGajA. The purified enzyme maintained both its nicking and degradative activities after storage in liquid form at 25 °C (room temperature) or 37 °C for 24 h and 48 h (Figure 5B,C). Even after storage for 7 days, LsGajA was still capable of efficiently degrading plasmid DNA, producing characteristic smear patterns on agarose gels (Figure 5D,E), demonstrating robust long-term stability at room and physiological temperatures. In contrast, LsGajA was sensitive to repeated freeze–thaw cycles. Although no obvious loss of activity was observed after five cycles, ≥10 cycles markedly weakened its ability to further degrade nicked or linear DNA into smaller fragments, indicating a pronounced reduction in non-specific nuclease activity and retention of primarily nicking activity (Figure 5F).

The non-specific nuclease activity of LsGajA suggests potential applications in molecular biology workflows, such as removing contaminating DNA during RNA purification or fragmenting DNA for chromatin immunoprecipitation assays. These findings indicate that repeated freeze–thawing should be avoided in practical use and that short-term storage at 4 °C or room temperature is recommended. Collectively, LsGajA exhibits exceptional thermal stability and maintains activity under common storage conditions, supporting its potential utility in molecular manipulation and biotechnological applications.

### 3.6. Gabija-Mediated Defense in Bacteria

**Figure 6:**
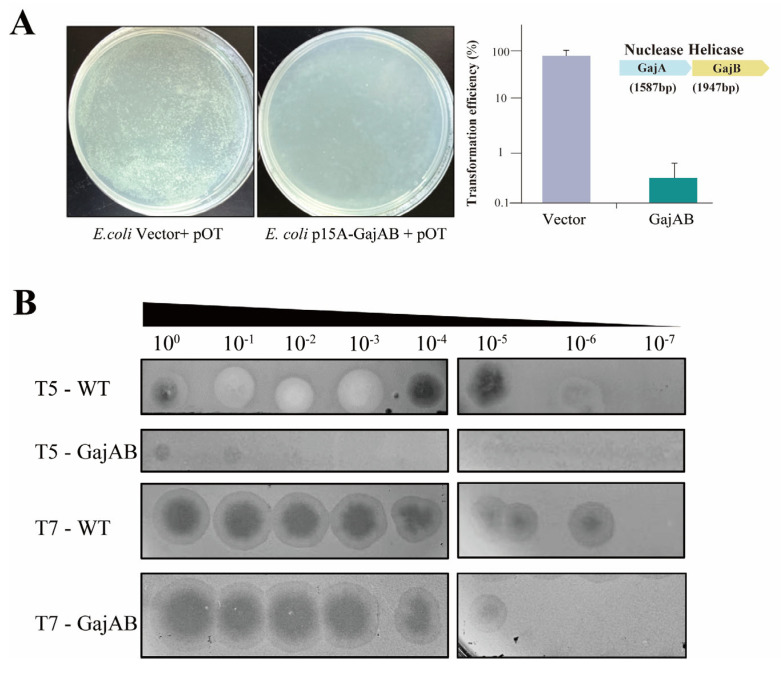
Gabija-mediated defense against exogenous plasmids and bacteriophages in *E. coli*. (**A**) Transformation assay of *E. coli* Top10 expressing the *L. salivarius* Ren Gabija system or empty vector (EV) with the plasmid pOT-RFP. Transformation efficiency was quantified to assess plasmid uptake. (**B**) Phage infection assay in *E. coli* BL21(DE3) expressing the Gabija system, with plaque formation evaluated for the bacteriophages T5 and T7.

To evaluate whether the Gabija system from *L. salivarius* Ren confers resistance to foreign nucleic acids in bacteria, we constructed an expression plasmid carrying the complete Gabija sequence (genomic coordinates: 1,336,359–1,340,554 bp) from the *L. salivarius* Ren and transformed it into *E. coli* Top10, with an empty vector as a control. The resulting strains were made competent and transformed with the pOT-RFP plasmid. Transformants were plated on dual-antibiotic agar, and colony numbers were quantified to assess the defensive effect of the Gabija system. If the Gabija system provides defense against exogenous plasmids, incoming plasmid DNA would be recognized and cleaved, preventing colony formation on a dual-antibiotic medium. *E. coli* expressing the Gabija system exhibited significantly lower transformation efficiency than the empty-vector control (Figure 6A), indicating that the Gabija system can recognize and degrade exogenous plasmid in a heterologous bacterial host.

We next assessed whether the Gabija system provides resistance against bacteriophage infection. The Gabija-expression plasmid was introduced into *E. coli* BL21(DE3), which was subsequently challenged with bacteriophage T5 (*Siphoviridae*) [35] and T7 (*Podoviridae*) [36]. Gabija-expressing bacteria showed strong resistance to T5, with plaque-forming efficiency (EOP) reduced by more than 10^4^-fold (Figure 6B). The plaques that did form were markedly fewer, smaller, and more diffuse, indicating potent inhibition of phage replication and spread. In contrast, resistance to T7 was weaker, with an approximately 10-fold reduction in EOP. These results indicate that Gabija-expression correlates with reduced susceptibility to certain bacteriophages in a heterologous *E. coli* system, with differential effects observed between T5 and T7.

## 4. Discussion

Gabija systems are recently identified bacterial defense systems that play important roles in resisting exogenous nucleic acid invasion and phage infection [17]. In this study, we provide the first systematic biochemical and functional characterization of a naturally occurring Gabija system from LAB, represented by the LsGabija system of *L. salivarius* Ren. LsGajA displays two distinguishable catalytic activities: the initial nicking activity that converts supercoiled DNA into linear or nicked intermediates, and the subsequent non-specific degradation activity that further cleaves DNA into small fragments. These two processes may rely on distinct DNA binding modes or catalytic states of LsGajA, consistent with the enzymatic properties of characterized GajA homologs from *B*. *cereus* [20]. Furthermore, we validated the defensive function of Gabija in vivo, thereby deepening the understanding of Gabija-mediated immunity and extending its functional scope beyond phage defense in LAB.

Our genomic analysis revealed that the Gabija system is present in approximately 9.3% of sequenced LAB genomes and is typically organized as a compact *gajA-gajB* operon. Notably, these loci are frequently located adjacent to canonical defense systems, such as restriction-modification (RM) modules, suggesting potential functional coordination. This organization is consistent with the concept of bacterial “defense islands,” in which multiple systems cluster to provide synergistic protection against foreign genetic elements [37,38]. Furthermore, the broad phylogenetic distribution of Gabija across diverse taxa supports the idea that horizontal gene transfer has played a significant role in its evolutionary dissemination.

Biochemical characterization demonstrated that LsGajA exhibits non-sequence-specific nuclease activity, efficiently degrading both plasmid and linear double-stranded DNA into heterogeneous fragments. This broad degradation profile differs from the activity reported for BcGajA, which has been mainly characterized as a nucleotide-regulated DNA-nicking endonuclease [21], suggesting that LAB-derived GajA homologs may possess lineage-specific catalytic properties. Importantly, DNA cleavage appears to proceed in at least two stages: initial nicking or linearization of supercoiled DNA, followed by extensive degradation into smaller fragments. Similar multi-step DNA processing behavior has been reported for other defense-associated nucleases [39], suggesting that flexible catalytic modes may be a common feature of prokaryotic immune effectors.

As a canonical two-component antiphage system, Gabija consists of the nuclease component GajA and the helicase component GajB [17]. LsGajB contains a conserved UvrD-like helicase domain, which is predicted to participate in DNA unwinding, substrate recognition, or the stabilization of the GajA-GajB complex [40]. In the characterized Gabija system from *B. cereus*, GajB forms a stable complex with GajA and modulates its nuclease activity for efficient antiphage defense [24]. Based on high structural and functional conservation, LsGajB is hypothesized to cooperate with LsGajA to promote the recognition and cleavage of invasive foreign DNA in lactic acid bacteria. If LsGajA and LsGajB were investigated together as a functional unit, the resulting activity might differ from that observed with purified LsGajA alone. For example, the LsGajA-LsGajB complex may exhibit altered cleavage kinetics; different preferences for supercoiled, nicked, linear, or potentially replication-associated DNA substrates; and a more physiologically relevant response to invading plasmid or phage DNA. LsGajB may also influence the activation threshold or substrate accessibility of LsGajA, thereby coupling nuclease activity to DNA topology, unwinding, or replication status. These possibilities may help explain why purified LsGajA displays broad non-specific nuclease activity in vitro, whereas the complete LsGabija system mediates defense against exogenous plasmids and phages in vivo. The detailed interaction and functional synergy between LsGajA and LsGajB warrant further biochemical and structural investigation in future studies.

A notable finding of this study is that LsGajA activity is influenced by DNA substrate form and host origin. Plasmids with identical sequences but derived from different *E*. *coli* strains exhibited markedly different susceptibilities to cleavage. Because these strains harbor distinct DNA modification systems—including methyltransferases and restriction-associated genes (e.g., *mcrA*/*mcrBC*, *mrr*, and *hsdRMS*) [41,42,43]—the observed variation is likely attributable to differences in epigenetic modification patterns, resulting in distinct plasmid modification states in vivo. However, this interpretation remains tentative, as factors such as DNA topology or co-purified proteins may also contribute. Future studies using precisely defined methylation-controlled substrates will be required to determine whether LsGajA directly senses DNA modification states.

The enzymatic properties of LsGajA further distinguish it from previously characterized homologs. Unlike BcGajA, LsGajA retains nicking activity in the absence of metal ions, whereas Mg^2+^ markedly enhances its random-cleavage nuclease activity. Sequence comparison with BcGajA indicates that LsGajA lacks several residues (N31, K35, and T36) present in BcGajA, which may contribute to its distinct Mg^2+^/Mn^2+^ response profiles and catalytic divergence, reflecting species-specific functional tuning within Gabija systems [21,34]. In addition, LsGajA exhibits apparent optimal activity at 45–60 °C and remarkable thermal stability, retaining partial activity even after exposure to near-boiling temperatures. While this property does not directly reflect the physiological temperature of *L. salivarius*, it is consistent with previous studies that report enzyme thermostability often arises from intrinsic structural features rather than environmental adaptation [44,45]. The broad temperature range, metal-ion response, and thermal robustness of LsGajA may reflect species-specific functional tuning within Gabija systems and may also increase its practical value as a robust DNA-processing enzyme. For example, LsGajA could be further explored for applications requiring sequence-independent DNA removal or fragmentation, while its thermal and storage stability may facilitate enzyme handling, transport, and use in industrial or field-compatible workflows. Nevertheless, its use in biotechnological contexts would require careful optimization of expression levels and application conditions to minimize potential off-target effects on target nucleic acids, engineered plasmids, or host genomic DNA, which may otherwise affect host viability or strain fitness. In addition, LsGajA activity may be host-dependent because intracellular nucleotide levels, metal-ion availability, DNA repair capacity, nuclease tolerance, and regulatory backgrounds can differ among bacterial species. Therefore, further biochemical, kinetic, and structural studies are needed to determine how these sequence differences affect metal coordination, catalytic activation, substrate cleavage, and the kinetic properties of LsGajA.

**Figure 7:**
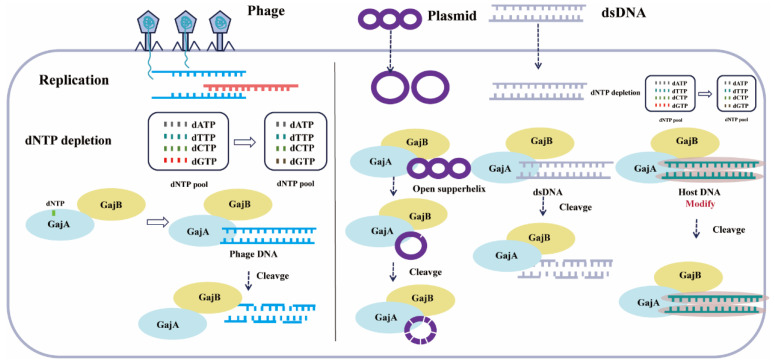
Model of LsGabija-mediated defense against invading phage, plasmid, or foreign dsDNA in LAB. Gabija senses foreign nucleic acids through dNTP depletion-dependent activation of GajA and cooperative action with GajB. Activated complexes target and bind foreign phages, plasmids and dsDNA; resolve supercoils into linear/nicked intermediates; and promote sequential degradation.

Importantly, we further confirmed that LsGajA activity is inhibited by dNTPs, consistent with the current model of Gabija activation [20,46]: under normal physiological conditions, high intracellular dNTP levels inhibit the activity of LsGajA. However, exogenous DNA invasion or phage replication depletes the dNTP pool, thereby relieving suppression and rapidly activating Gabija for an “intrusion-responsive” defensive reaction (Figure 7). Functionally, the Gabija system significantly reduced plasmid transformation efficiency and conferred strong resistance to the bacteriophage T5 but only moderate resistance to T7. This difference may be associated with their distinct infection and replication strategies. T5 delivers its genome through a two-step transfer process, in which the first-step-transfer DNA is injected and expressed before the remaining genome enters the host cell [47,48]; additionally, T5 does not encode its own RNA polymerase and is therefore more dependent on host transcription during early infection [47]. These features may extend the period during which incoming phage DNA is accessible to Gabija-mediated DNA nicking/cleavage and nucleotide-depletion-associated antiviral defense [20]. In contrast, T7 rapidly switches from host RNA polymerase-dependent early transcription to phage-encoded T7 RNA polymerase-driven transcription [49], while suppressing or modulating host RNA polymerase activity and initiating efficient DNA replication [49,50]. This rapid phage-controlled infection program may limit the opportunity for Gabija-mediated DNA surveillance, thereby allowing for partial escape from Gabija immunity. Thus, the stronger anti-T5 activity of Gabija likely reflects differences in genome injection kinetics, host-transcription dependence, and replication dynamics between T5 and T7.

## 5. Conclusions

In conclusion, this study provides the first systematic biochemical and functional characterization of a naturally occurring Gabija system from LAB. LsGajA from *L*. *salivarius* Ren functions as a robust nuclease with broad non-sequence-specific dsDNA degradation activity, substrate-dependent differences in DNA cleavage efficiency, dNTP-mediated inhibition, and remarkable thermal and storage stability. The complete LsGabija system also reduces exogenous plasmid acquisition and provides resistance against bacteriophages in a heterologous host.

Given its potent nucleic acid-degradation activity and streamlined genetic architecture, Gabija exhibits considerable potential for applications in synthetic biology and applied microbiology, such as enhancing probiotic phage resistance, improving industrial strain genetic stability, limiting horizontal gene transfer, or serving as a molecular tool for non-specific DNA clearance and DNA fragmentation. Future studies dissecting the cooperative mechanism of LsGajAB and its native regulatory dynamics will further advance our understanding of this emerging defense system and support its optimization for biotechnological applications.

## Figures and Tables

**Table 1:** Sources of bacterial strains containing the Gabija system.

Strain	Isolation Source
*L. salivarius* Ren	The feces of a healthy centenarian
*L. salivarius* JCM1046	Swine intestine
*L. casei* 21/1	Corn silage
*L. paracasei* Lpp123	Artisanal dairy
*L. reuteri* TMW1.112	SER sourdough
*L. brevis* DmCS003	*D. melanogaster* gut
*L. salivarius* UCC118	The terminal ileum
*L. plantarum* SF2A35B	Sour Cassava, South America
*L. rhamnosus* GG	Human gastrointestinal mucosa
*L. lactis* A12	A traditional French wheat sourdough
*L. lactis* KLDS 4.0325	Homemade koumiss
*L. lactis* Bpl1	Wild-Caught *D. melanogaster*
*L. lactis* ATCC19435	Milk (dairy starter)
*B. cereus* VD045	Soil, Greenland
*B. cereus* HuB5-5	Soil, Belgium
*E. coli* E24377A	Tribal drinking water source in India
*E. coli* UTI89	Acute Cystitis isolate

## Data Availability

The original contributions presented in this study are included in the article/Supplementary Materials. Further inquiries can be directed to the corresponding authors.

## Supplementary Materials

The following supporting information can be downloaded at: https://www.mdpi.com/article/10.3390/microorganisms14061353/s1, Table S1: Plasmids used in this study; Table S2: Oligonucleotides used in this study; Table S3: Statistical table of LsGajA restriction enzyme cutting site sequences; Data S1: Nucleotide sequences of LsGajA and LsGajB; Figure S1: Analysis of GajA and GajB junction; Figure S2: AlphaFold-predicted structural models of LsGajA and LsGajB; Figure S3: Western blot analysis of LsGajA; Figure S4: Analysis of GajA cleavage sites.

## Abbreviations

The following abbreviations are used in this manuscript:

Abi	Abortive infection
AbiEii	Abortive infection system Eii
dATP	Deoxyadenosine triphosphate
dCTP	Deoxycytidine triphosphate
dGTP	Deoxyguanosine triphosphate
dNTPs	Deoxynucleoside triphosphates
dsDNA	Double-stranded DNA
dTTP	Deoxythymidine triphosphate
EOP	Efficiency of plating
EV	Empty vector
HGT	Horizontal gene transfer
IPTG	Isopropyl β-D-1-thiogalactopyranoside
LAB	Lactic acid bacteria
LsGajA	The GajA of *L*. *salivarius* Ren
LsGabija	Gabija system of *L. salivarius* Ren
PFU	Plaque-forming unit
RM	Restriction modification
SDS-PAGE	Sodium dodecyl sulfate–polyacrylamide gel electrophoresis
SEM	Standard error of the mean
TA	Toxin–antitoxin
TOPRIM	Topoisomerase-primase
UTR	Untranslated region
Wadjet	Wadjet defense system
