# Supplementary material for: Biochemical Characterization of LsGajA: A Key Nuclease for Gabija Defense in Lactic Acid Bacteria
Source: Microorganisms. 2026 Jun 16;14(6):1353. doi: 10.3390/microorganisms14061353 (PMC13304284; doi:10.3390/microorganisms14061353)
Supplement: Supplementary file 1 [file microorganisms-14-01353-s001.zip › microorganisms-4311585-supplementary.pdf]

Biochemical Characterization of LsGajA: A Key Nuclease for Gabija Defense in Lactic Acid Bacteria

Kexin Li, Yujing Tian, Juyue Luo, Shiyu Ma, Jinhai Huang, Lei Zhang * and Deping Hua *

School of Life Sciences, Tianjin University, Tianjin 300072, China; 13920967339@163.com (K.L.); tianyujingw@tju.edu.cn (Y.T.); ljyjy525@163.com (J.L.); mashiyu@tju.edu.cn (S.M.); jinhaih@tju.edu.cn (J.H.)

* **Correspondence:** zhanglei@tju.edu.cn (L.Z.); huadeping@tju.edu.cn (D.H.)

TABLE S1

Plasmids used in this study.

Plasmid	Description	Notes
pET-28a-GajA	6.9kp; kanR; recombinant pET-28a plasmid carrying the LsGajA coding sequence under the T7/lac promoter for heterologous expression of His-tagged LsGajA in *E. coli*.	Constructed in this study
p15A-Cm-repDE-GajA/B	9.6kb; cmR; carries the *LSGajA-LSGajB* operon under its native promoter, used for co-expression of both genes in *E. coli*.	
pJW	8.3kb; ampR(in *E. coli*) and emR(in LAB); *E. coli*-LAB shuttle vector; used as a substrate plasmid for LsGajA cleavage assay.	Maintained in our laboratory
F1315	4.9kb;cmR; *E. coli*-LAB shuttle vector; used as a substrate plasmid for LSGajA cleavage assay.	
pot-PFR	6.1kb; ampR; URA3 marker (in yeast); *E. coli*-yeast shuttle plasmid; contains an RFP transcription unit; used as a substrate plasmid for LSGajA cleavage assay.	
pGM-T	5.0kb; ampR; TA cloning vector; used as a substrate plasmid for LSGajA cleavage assay.	
pBTs	6.9kb; kanR; cloning vector; used as a substrate plasmid for LSGajA cleavage assay.	
pUC19	2.7kp; ampR; standard cloning vector with lacZα for blue-white screening; used as a substrate plasmid for LSGajA cleavage assay.	
pET-28a	5.4kb; kanR; empty expression vector, IPTG-inducible T7/lac expression vector with N-terminal 6×His tag; used as a substrate plasmid for LSGajA cleavage assay.	
pLC-DE	8.2kb; cmR; *E. coli*-LAB shuttle vector shuttle vector with replicons for both *E. coli* and *Lactobacillus*; used as a substrate plasmid for LsGajA cleavage assay.	

TABLE S2

Oligonucleotides used in this study.

Oligonucleotide	Sequence (5'→3')	Notes
GAJA+B-F	CTTTTTGTGCGGACCTGCAGCTTCAGTATCA ACTCCGTGACACAAC	Gene amplification and plasmid construction
GAJA+B-R	CTTATTAATCAGATAAAATATTTCTAGAGGT TGCCATAGTCTCACTTCCTATACC	Gene amplification and plasmid construction
DE-GAJA+B-F	GGTATAGGAAGTGAGACTATGGCAACCTCT AGAAATATTTTATCTGATTAATAAG	Reverse amplification of the plasmid vector
DE-GAJA+B-R	GTTGTGTCACGGAGTTGATACTGAAGCTGC AGGTCCGCACAAAAAG	Reverse amplification of the plasmid vector
GAJA-F1	CGCGGATCCGAATTCGAGCTCATGAAAAGA TAAAAC	Plasmid construction
GAJA-R1	CGCAAGCTTGTCGACGGAGCTCACCTACTT CATCAACCATTCCAAACCTCC	Plasmid construction
1315-F1	CTTGAAGTCATGCGCCGGTTAAGG	Amplification of the dsF-1315F fragment
1315-R1	AAAATCTCACTCACTGGAAAAATCTCAC	Amplification of the dsF-1315F fragment
1315-F2	AGTATGATTATAACATAGTATTTCAATAAG GTTCAAC	Amplification of the dsF-1315R fragment
1315-R2	GCGATATCATGCGCATGCAAGC	Amplification of the dsF-1315R fragment
pJW-F1	GGCCAGTGCCAAAGCTTGC	Amplification of the dsPJW1 fragment
pJW-R1	CCTTCTATTTCGGTTGGAGGAGGC	Amplification of the dsPJW1 fragment
pJW-F2	CCTTGAGCCAGTTGGGATAGAGC	Amplification of the dsPJW2 fragment
pJW-R2	CTGTGGATAACCGTATTACCGCC	Amplification of the dsPJW2 fragment

pJW-F3	CATGGTCATAGCTGTTTCCTGTGTG	Amplification of the dsPJW3 fragment
pJW-R3	GCTTAAGCTGCCAGCGGAATGC	Amplification of the dsPJW3 fragment
M13F	CGCCAGGGTTTTCCCAGTCACGAC	Verification of the recombinant plasmid
M13R	AGCGGATAACAATTTCACACAGGA	Verification of the recombinant plasmid
PGAJA+B-F	CTTCAGTATCAACTCCGTGACACAAC	Verification of the recombinant plasmid
PGAJA+B-R	GGTTGCCATAGTCTCACTTCCTATACC	Verification of the recombinant plasmid
PGAJA-F	CTTCAGTATCAACTCCGTGACACAAC	Verification of the recombinant plasmid
PGAJA-R	CAGCAGGCGCATTCACAAG	Verification of the recombinant plasmid
PGAJB-F	CTAGGACTCTTGAAGAAGCGATGC	Verification of the recombinant plasmid
PGAJB-R	GGTTGCCATAGTCTCACTTCCTATACC	Verification of the recombinant plasmid
T7	TAATACGACTCACTATAGGG	Verification of the recombinant plasmid
T7-TER	TGCTAGTTATTGCTCAGCGG	Verification of the recombinant plasmid

TABLE S3

Statistical Table of LsGajA Restriction Enzyme Cutting Site Sequences.

**Statistical Table of LsGajA Restriction Enzyme Cutting Site Sequences**			
F1	UP：5'-TATAAC↓ATAGTA-3'<br>DOWN：5'-GAAGTC↓CATGGA-3'	F2	UP：5'-TATGCC↓TCCTAA-3'<br>DOWN：5'-CTCACA↓AAAATC-3'
F3	UP：5'-TAATTG↓GTGGAC-3'<br>DOWN：5'-TTTATC↓TTCCTT-3'	F4	UP：5'-ATGAGG↓GTGTCA-3'<br>DOWN：5'-CCGGTG↓CGTCAG-3'
F5	UP：5'-TATGGC↓CGCGTT-3'<br>DOWN：3'-TATCGC↓ATCTGC-3'	F6	UP：5'-CCTGAA↓GTCAGC-3'<br>DOWN：5'-TATCGC↓ATCTGC-3'

Note: down arrows mark the nicking site

Data S1

Nucleotide sequences of LsGajA and LsGajB

LsGajA:

ATGGATGAAAAGATAAAACATAATATTGAGCTAAAAGAAAGATATCTAAAGCAAAGAATAG
TTACGTTATATGTCAATTCAATTGTTCCAACATGCTATTTTTGCGATGAAAAGTATGAGAATA
GATGCCAAATGGATGATGTAAAGCAGTTTAGGAATCTATTTAACTTTTGCTTTATTAAAGCAA
GTAGACCTTTAGATGATGATTTATCAGACCATTCTCATTCGATCAGTAAACAGATGATTAAAA
TGGCTAAATTAGATGGAGAATGGAATGAATTGATAGATAAATTACCTGATGAAATTTTAAAG
CCTATTCAAGACAAAGATATCAGTAAGAAGGTTCAGGAAACGTCGCTAAATTCATTAAAAG
AGACAATTATGGCTATTGAAGAAACCAATGGTGGGAGATCAGGTGAATTGATGTTGGATATG
CTTGTTACAGAAGAAGATATTAGTGATTTACTTCAAAGGATCACAATGGCTACGTATTGTGTC
GATGGTTATTTCCTAGGTGAAGAATCACAAGGATTAGGCTATAGCAATATGATATATATACATC
TTCAGTTAAATGAATATGAAAATAGTAAAGATAGCTGTAAGGTAAATGTTTTCTTTGTTGAA
GAACCAGAATCACATATGCATCCTCAAATGCAGCAAGTATTTATCAAATATCTCATTGAACAT
TATAAAAATGGTATTCAAGGATTAATTACAACACACTCAAATGAAATGGTGCGAGTTGCTGG
GATAAAACATTTACGTGTTATTAGAAAAACAGATAGTTTTTAAGTGAGCTCCATGATCTTTC
GAAATTAATCAATGAATTACAAGAATCAAGTAATTCAGAGGATAAGCTATTAGCCAATTTCTA
TGATTGGTTTTTTGAGATTGGTTATTCTGAACTTATATTTGCAGATAAAGCTATTTTTTATGAA
GGAGATACAGAACGATTATATATCAGAAAGCTTTTAACATTAAAAAAATATGAAAAGCTAAA
GCAACAATATATTGCTTATATTCAGGTTGGAGGGGCATATGCAAAAAATTATCAAAAAATGAT
AGAGTTGTTAGGTATCAAATCATTAGTCATTACTGATATAGATTATTCTAAAGATGCAGAAGT
AATAGATGAGATAGAAAGTTCTGAAATAACTAATGCGACAATAAAGGCGTTCTATAGGATTG
ATAACCCTGAGAGCAATCCAACAGTTAAAGATCTATATGAATGGAAAAATAAGAATAAGAAT
ATTATAAGTAATGGGTTGATTTATACTTGCTTTCAAACTAACAATGATGGGTATTCTAGGACT
CTTGAAGAAGCGATGCTTTCGAAGTATTTCTCGATTGATGTTACTACAAGTCTTACCAAAAG
AGAATGGATAGAAAAAAAAGAAAATTCAAAACTAAAATTTGTTATTCCAACTAAAGGAATA
GAAGAAGAAAATTCTATCAAGTTGCGAGATATTTTAAAATCGACATCTAGTTCAAAAACAA
ATTTTATGTATTCTGTAGTACTTAATGAAAAGGTAGAAAAAACTGAACCTAATTATATTCAAG
GAGGTTTGGAATGGTTGATGAAGTAG

LsGajB:

ATGGTTGATGAAGTAGAGAATATTTATCTTGTGAATGCGCCTGCTGGAAGTGGTAAAACTAC
ATGGATCCGTAAACAGGTTGAAAAACATTTGCTTGAAAATGATGACGATAATATTTTATGTAT
AACATATACAAATCGAGCTGCAGAAGAATTGGGAAGGGATATAAAGTCTAATAGAGTTTTTT
TTGGAACAATTCATAGTTTTATAAGTAACTATATGGGAAGTTTTTTTGGACATAAAGAGATAA
TTGATCTATATTGGGAACTATACGAGGAGAAAATAGCACAAAGAATTGCTAATGTTGAAAAC
AAGAAACATGTTTCAATTAGTAATGAAAGATATATAGAAAAATACGGAGTACTGGACATGGA
AACAGTGCACTCAAATTTGAAGAAGATAAGTTATGGTGAAACTCAATTCACATCGTTGTATT
ATGGATTATTAAGCCATGATGACTTACTATCGTTCACCAAGAAGGCAGTTGAAAGATATCCT
GTTATTCTTAAAAAAATTCGTGACAAATATCAACTTGTTTTTATAGATGAGTACCAGGATACA
AATGCGGATATTCTTAAATTGTTTTATTCATCGATGGTGTCAGGTAAGGGAAAGTTGTATTTA
TTAGGTGACAAAATGCAACAAATATATAAGAACTACGATGGGAGCTTTGAAGATACTTTCAA
TATATTGAATAAGTCTACGAGACTAGATGTTAACTATAGAACGACTCCGTATATTGTCAATATT

TTGAATGCTATATACAATGATAAATCATTACAACAATATCCTTATGATAAAAATTTAGATAGTC
AAATGTTGTTTAAGCCTAAGGTTATTTTTACAAATGATAGAGATAAGACAGTTAATATATTTA
CAAAGAAAAATAAAGGTACCTTGGTTCTGTATTTAACAAATAAAGAACGTTTTTATGGTATT
GGTGTAAAAAAACTTTATGAGTCATTTAATAAAATACCTAAGTATCAATTTACAGGGAAGTAT
AGTGTTGTTGATGTGCTTACAAAGGAAGAGGTTTGGAATCAAGATATTCTTTTGAGTATAAT
TTTTTTACTCATTGATATTTGTGATTATTATATTAATGGTAAGATAGGAAACATTTTTAAGATAA
CGCGGAACAATGAAAAAATTTTTAATAGAAAAGTTTTTTCAATACGATTCCATGCTGATAAA
AAAGCATTGCATGATAAATTAGATAATTTAATTGCTATTTACCAAGAAGAAGGAACTACTATA
GGTTCATTCCTTAAGAGTTGTTGTGATAATAGACTTATAGAAGAAGAGTACTATGGAGAAAT
TATTGAGGATGATGACTATGCTTCCGCATTGGAGGTTACGCTATCTGAAATATTAATTTTAAG
GAATTTTTTAGAAAATCCATATATTTCGACGCAACATGGTGTTAAGGGAGAAAGTCATGATA
CTGTTCTGTTTGTGGCGGAAAATAATTCTAAAATACCTATAGTGAATATGAGCAAATTCTTTG
ATCTATGGAGTAAAGTGAATGTAGTTCTAAGAGATTTTGATAATTTTTATTATGCTTATTTAAA
AATGATTAAGAGTATTGAAAAGAAATGTGGTATGAAAATATCAAAGATAAATTCGAATGATT
ATAGAAAAAAATGAAACATTTATTTCAGAAAAAAATATCAAAATTTAACGAGATATATTATGATA
ATGACTATTATATTGTACTTTTAAAAGATAAATTTGATAAGTATTTTGTTAAAAAGAATAACAA
AAGTGCACAAGAATGCTTGAAAGAGAATGCAGTTTATGGTGCTTTGGTTGCTTATCGACTTT
TTTATGTTGGATGTTCTAGAGCACGTAAAAATCTTGCAATTGTAATTAACAATGCTGATGTAG
TTGCTTTTGAAGAACGATTAAAGAACAAATTTAAAGATATTGGTTTTGAAATTGAGGAAATT
TAA

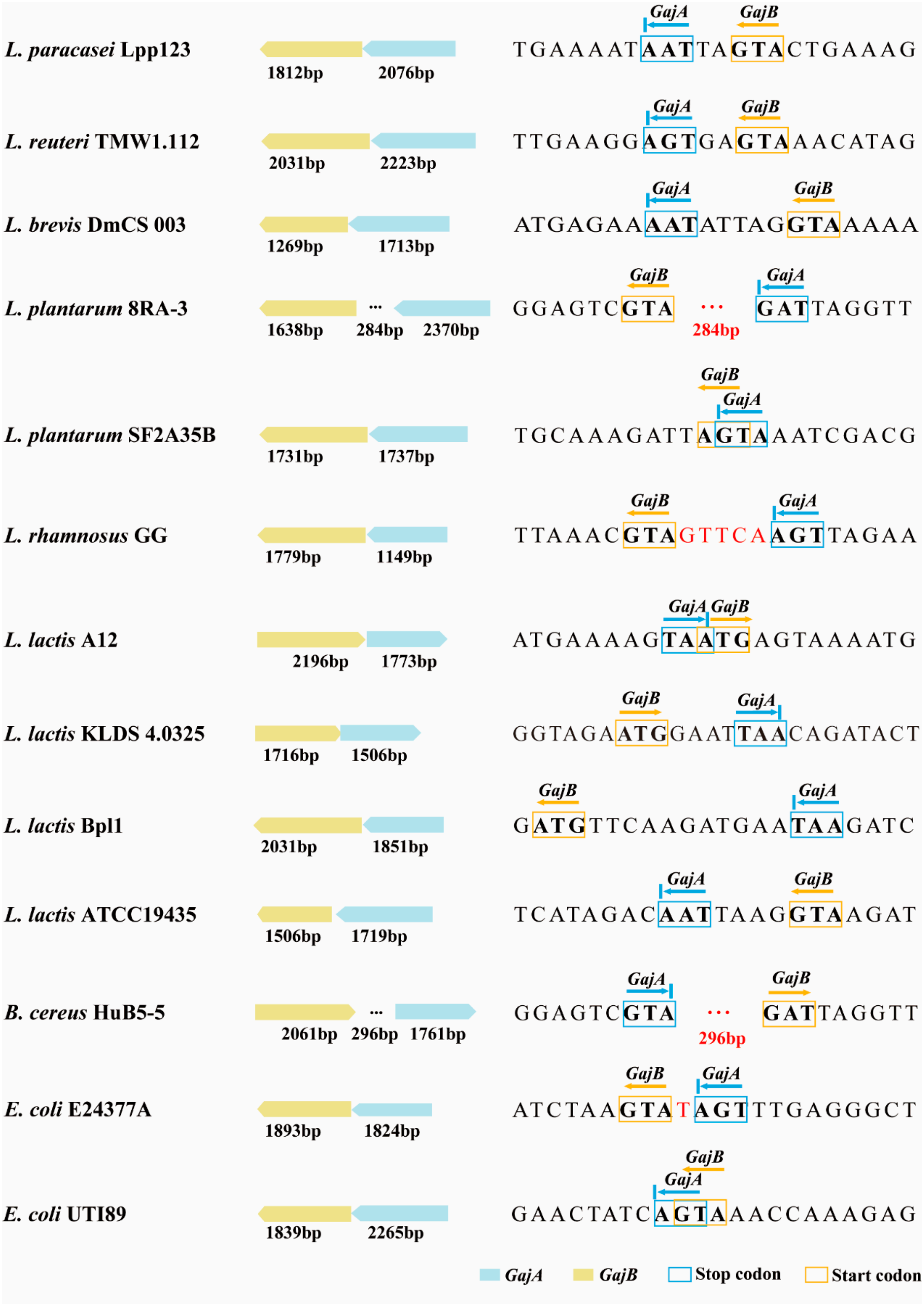

Figure. S1. The analysis of *GajA* and *GajB* junction, with the core genes *GajA* and *GajB* forming a compact cluster.Right, enlarged nucleotide sequences at the *GajA*–*GajB* junctions. Blue and yellow boxes indicate stop codons and start codons, respectively. Arrows above the sequences indicate the transcriptional orientations of *GajA* and *GajB*. The red markings indicate the intergenic sequence

between *GajA* and *GajB*.

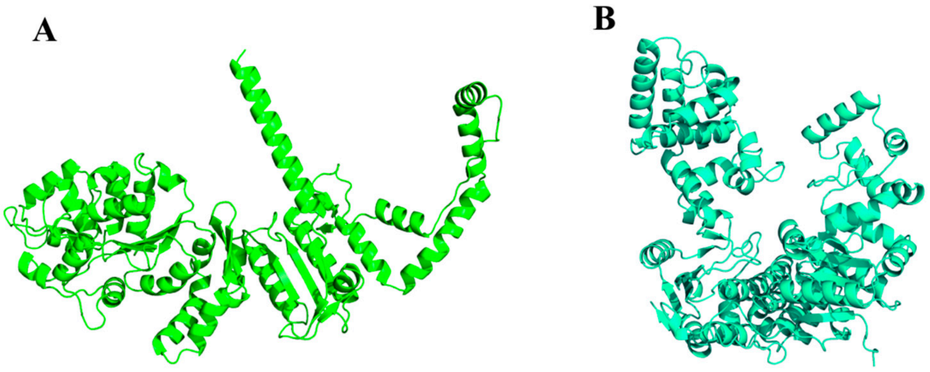

Figure. S2. AlphaFold-predicted structural models of LsGajA and LsGajB. (A) Predicted structure of LsGajA. (B) Predicted structure of LsGajB

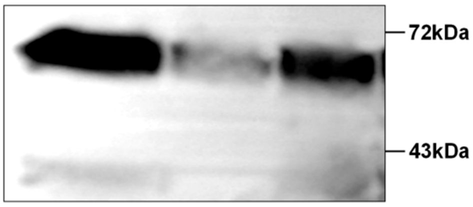

Figure. S3. Western blot analysis of LsGajA. Anti-His western blot analysis mainly detected a His-positive signal corresponding approximately to full-length LsGajA.

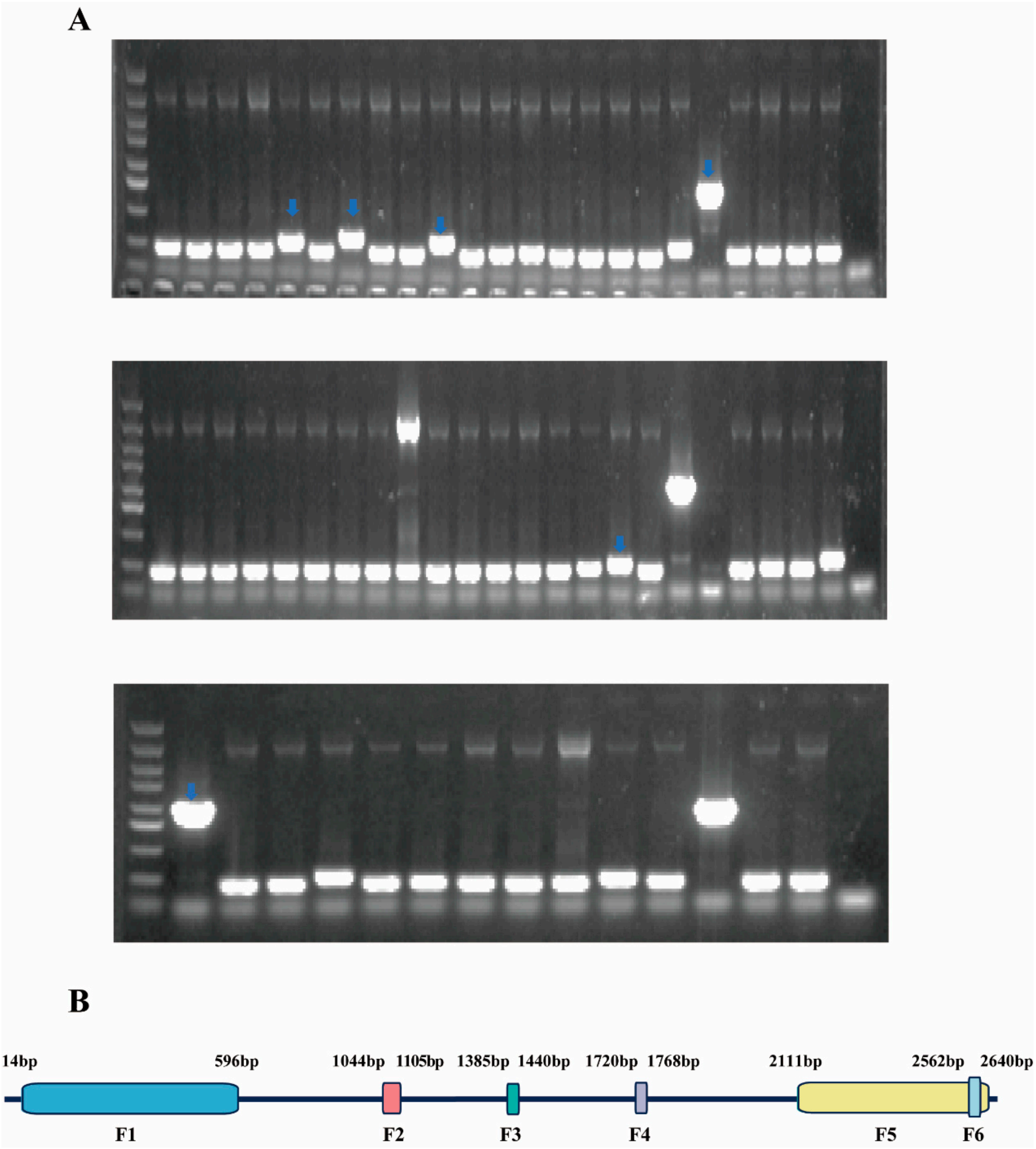

Figure. S4. Analysis of GajA cleavage sites. (A) Colony PCR analysis of *E. coli* clones transformed with the digested dsF1315R products cloned into a plasmid vector. Clones exhibit highly variable insert lengths, indicating random cleavage. (B) Schematic representation of the positions of cleavage products within the F-1315R fragment.
